# The impact of social vulnerability on primary vaccine coverage in children with sickle cell disease

**DOI:** 10.3389/fpubh.2025.1689797

**Published:** 2026-01-05

**Authors:** Jiajing Scarlette Shi, Erin LaFon, Ankit Sutaria, Brandon Kyle Attell, Mei Zhou, Amy Tang, Angela B. Snyder

**Affiliations:** 1 Georgia Health Policy Center, Andrew Young School of Policy Studies, Georgia State University, Atlanta, GA, United States; 2 Department of Public Management and Policy, Andrew Young School of Policy Studies, Georgia State University, Atlanta, GA, United States; 3 Jimmy and Rosalynn Carter School of Public Policy, Georgia Institute of Technology, Atlanta, GA, United States; 4 Georgia Department of Public Health, Atlanta, GA, United States; 5 Aflac Cancer and Blood Disorders Center, Children’s Healthcare of Atlanta, Atlanta, GA, United States; 6 Emory University School of Medicine, Atlanta, GA, United States

**Keywords:** immunization, pediatric, sickle cell disease, socioeconomic characteristics, vulnerability

## Abstract

**Introduction:**

This study examines the relationship between social vulnerability and up-to-date (UTD) primary vaccine coverage among children with sickle cell disease (SCD).

**Methods:**

This retrospective cohort study included children with SCD born in Georgia between 2008 and 2019, identified through the state newborn screening program. Immunization records were obtained from the state registry, and birth addresses were matched to census tract level Social Vulnerability Index (SVI) scores. Children were considered UTD if they completed the recommended vaccine doses by 24 months. Multivariable logistic regression assessed the association between overall and subtheme SVI scores and vaccine completion, adjusting for demographic and clinical covariates.

**Results:**

The study included 1,337 children with SCD. Of these, 37% lived in areas with the highest SVI vulnerability, and 12% lived in the least vulnerable areas. Overall, 58% of children were UTD with their primary vaccine series. Children with moderate or high vulnerability in the socioeconomic subtheme had significantly higher odds of having UTD poliovirus vaccine, measles, mumps, and rubella vaccine, *haemophilus influenzae* type-b vaccine, and hepatitis B vaccine compared to children with low vulnerability. Higher vulnerability in the housing type and transportation marginally decreased the odds of completing the diphtheria, tetanus, and acellular pertussis vaccine.

**Discussion:**

Children with SCD are disproportionately concentrated in areas of high social vulnerability. The SVI can help identify neighborhoods for targeted vaccine outreach, especially in communities with high housing and transportation vulnerability. Efforts should prioritize multi-dose vaccines and the varicella vaccine.

## 1 Introduction

Sickle cell disease (SCD) is a genetic blood disorder that affects approximately 7.74 million individuals globally (1). In the United States, the SCD prevalence is 100,000 individuals in the United States, with the majority of those affected being non-Hispanic Black or African American (2, 3). Children with SCD may begin to experience symptoms of hemolytic anemia and vaso-occlusion as early as 5 months of age (4–7). Progressive vaso-occlusive damage to the spleen leads to immune deficiency in children with SCD, increasing their susceptibility to infections by encapsulated organisms, which can result in severe morbidity and mortality (8–12).

Preventive immunizations have been effective in protecting children with SCD from severe infections and have substantially improved survival into adulthood (13). Following the licensure of the pneumococcal conjugate vaccine, the incidence and mortality of invasive pneumococcal disease declined significantly among children with SCD (14). Notably, rates of invasive pneumococcal disease among children with SCD under 5 years of age dropped by approximately 90%, and related hospitalizations declined nearly 3-fold (15, 16). The Advisory Committee on Immunization Practices from the U.S. Centers for Disease Control and Prevention recommends all children complete the primary vaccine series by 24 months of age; completion is especially important for those with SCD (17). This series consists of 7 vaccines: diphtheria, tetanus, and acellular pertussis (DTaP) (4 doses), poliovirus (3 doses), measles, mumps, and rubella (MMR) (1 dose), *haemophilus influenzae* type-b (3 or 4 doses depending on product type), hepatitis B (3 doses), varicella (1 dose), and pneumococcal conjugate (4 doses of 7-valent or 13-valent vaccine) (17). For children with SCD, staying up to date with the primary vaccine series is especially critical since additional pneumococcal vaccines (PPSV23 or PCV20) are recommended after completion of the primary series to provide additional protection (14, 18, 19).

Despite the importance of preventive care, immunization coverage among children with SCD remains suboptimal (20–25). Prior studies indicated that less than 70% of children with SCD had completed the primary vaccine series by 35 months of age (22, 25). Coverage rates for the MMR vaccine and the DTaP vaccine among children with SCD are below the 90% coverage goal outlined in Healthy People 2030 (26). Notably, pneumococcal and varicella vaccination rates among this population are below 76% (25).

Existing literature on factors influencing immunization coverage in children with SCD is limited and primarily focuses on healthcare provider-related factors. For instance, Bundy et al. (27) found that more frequent hematologist visits were associated with higher influenza vaccination coverage. A hospital-based intervention study also suggested that improved care coordination and stronger linkages between services can help boost vaccination rates (28). While such quality improvement initiatives at the health system level may help improve vaccination coverage, there remains a significant gap in understanding how patient-level and community-level characteristics shape systemic barriers to timely vaccination. One study found that a population-based recall system targeting parents was more effective and cost-efficient in increasing primary series immunization coverage than practice-based interventions focused on primary care providers (29). This knowledge gap restricts the ability to design large-scale policy interventions and implement programs that effectively reach underserved populations. Moreover, existing research has primarily focused on barriers to influenza vaccination, with far less attention given to the primary vaccine series, which is equally important for children with SCD.

Community-level social vulnerability refers to demographic, socioeconomic, and other community-level stressors that can adversely affect populations during hazardous events (30). Economically disadvantaged communities often experience higher rates of infectious disease due to hazardous environmental exposures, overcrowding, and reduced access to healthcare (31–36). Limited social networks and fewer resources can further hinder the ability of residents in these communities to seek care during outbreaks (31, 36, 37). Research indicates that social vulnerabilities not only affect communities’ susceptibility to disease outbreaks, but also shape the use of preventive health services, including vaccination (38–40). In recent years, the Social Vulnerability Index (SVI) has emerged as a widely used metric to assess community-level vulnerability in health services research (41, 42). While much of this research has centered on COVID-19 immunization, which has found that higher social vulnerability is associated with lower vaccine uptake, similar patterns have been observed for influenza and tetanus-diphtheria-acellular pertussis vaccinations (40, 43–46). Marginalized communities, such as those affected by SCD, are disproportionately susceptible to poor health due to structural inequities (47). However, only a handful of studies have examined the role of social vulnerability within the SCD population. Two studies found that a higher SVI score was associated with increases in emergency department visits (48, 49). Another study (50) found a link between higher SVI and increased mortality in the SCD population.

Given the limited availability of socioeconomic data in SCD research, the SVI offers a practical and informative proxy for assessing community-level disadvantage for this population. For children with SCD, it is especially important to examine how neighborhood-level vulnerability impacts vaccine uptake, since they are disproportionately affected by socioeconomic hardship (2, 51, 52). By examining the relationship between community-level social vulnerability and vaccine coverage for this at-risk population, large-scale interventions and policies could better target communities with need and leverage resources to help boost preventive health care utilization. This study examines the relationship between social vulnerability and primary vaccine coverage among children with SCD in Georgia, United States.

## 2 Materials and methods

### 2.1 Data sources

This study identified children with SCD born in Georgia between 2008 and 2019 through the Georgia newborn screening program (NBS) and obtained these children’s immunization records up to 24 months of age from the state’s immunization registry. The detailed process of matching children’s immunization information is explained elsewhere (25). The Georgia State University Institutional Review Board considered this study exempt because it was conducted as a secondary analysis of data collected for the public health surveillance of hemoglobinopathies (IRB# H11142) under an active agreement between Georgia State University and the Georgia Department of Public Health.

### 2.2 Measures

#### 2.2.1 Child outcomes

The study created 2 outcome measures for up-to-date (UTD) status on vaccines, UTD for a single vaccine and UTD for the entire primary series. Children with SCD were considered UTD for a vaccine if they completed the recommended doses for that vaccine by age 24 months (17). Children were considered UTD for the primary series if the children completed all the recommended vaccines in the primary series and their related doses by age 24 months. If a child missed any vaccine dose as of 24 months of age, they would not be considered UTD.

#### 2.2.2 SVI

The study used the SVI developed by the Centers for Disease Control and Prevention (30). The SVI has 4 themes that, when combined, create a composite score for community social vulnerability. These themes include socioeconomic status, housing type and transportation, household characteristics, and racial and ethnic minority status (30). These 4 themes are composed by using 15 variables from the American Community Survey (30) before 2020. The socioeconomic status theme includes variables indicating below 100% poverty, unemployed, income, and no high school diploma (53). The housing type and transportation theme includes multi-unit structures, mobile homes, crowding, no vehicle, and group quarters. The household characteristics theme includes aged 65 or older, aged 17 or younger, civilian with a disability (except for the 2010 SVI), and single-parent household. Lastly, the racial and ethnic minority status theme includes racial minority and speaks English “less than well.”

The SVI ranges from 0 to 1, with higher values representing greater social vulnerability. Each census tract in Georgia receives an SVI score which ranks them from the area with lowest social vulnerability to the highest. For this study, the overall SVI and the four subtheme scores were categorized into three levels, with 0–0.25 being the lowest vulnerability level, above 0.25 to 0.75 being the moderate vulnerability level, and above 0.75 to 1 being the highest vulnerability level (40, 52, 54, 55). The study combined the 2 middle quartiles due to the limited number of observations in the SCD population to improve statistical power.

**Figure 1:**
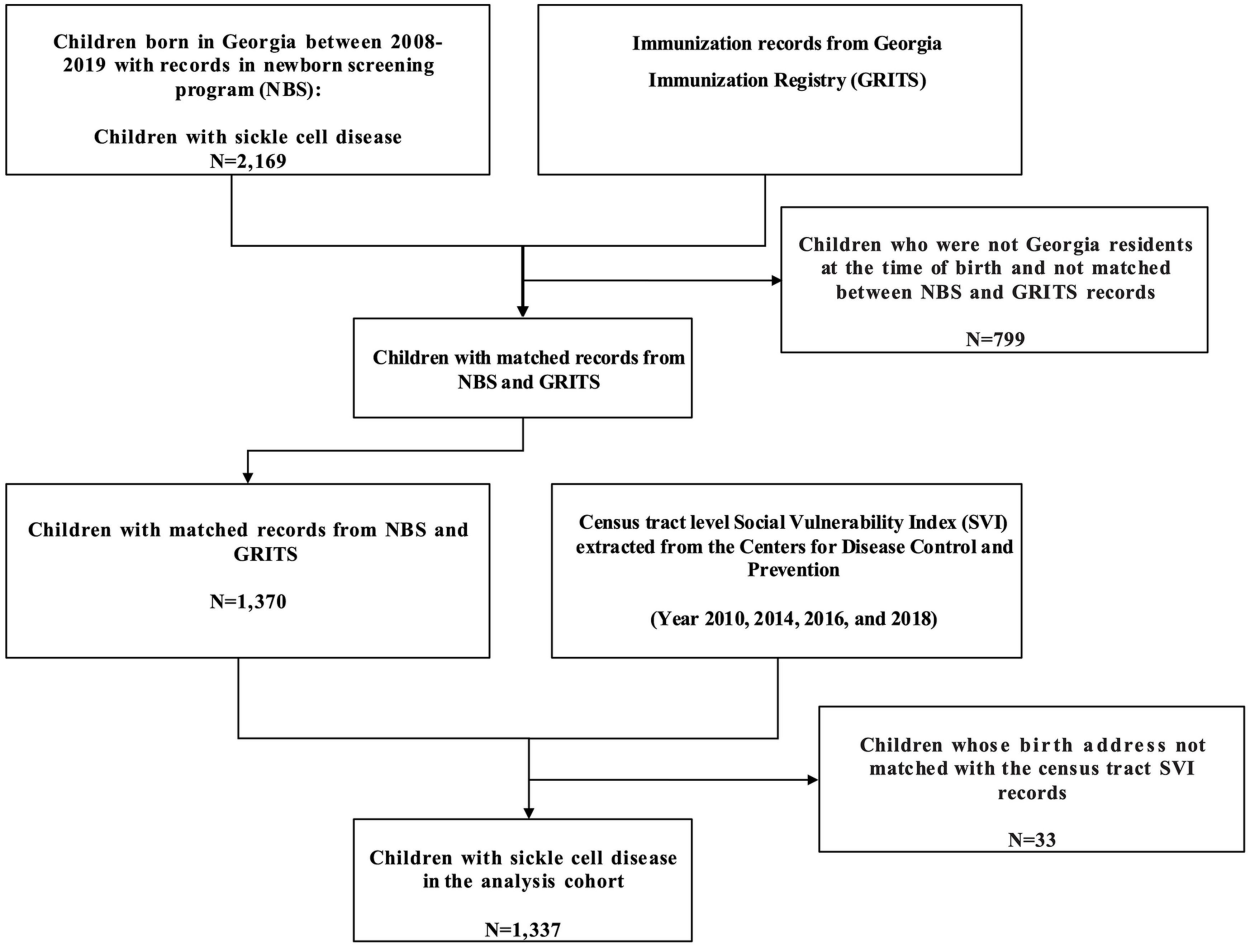
Data linkage.

This study utilized SVI scores from 2010, 2014, 2016 and 2018. These years were selected because they closely aligned with the study period (2008–2019) and represented the social vulnerability of children’s residential areas from birth to 24 months. These 4 years of SVI data are more comparable than other years since there are fewer changes in the underlying source data and census tract boundaries during their creation (53). The study assigned each child with a single year SVI score based on the proximity of SVI year and their birthing year. Specifically, children born between 2008–2012 were assigned the 2010 SVI scores. The 2013–2014 birth cohort was assigned the 2014 SVI scores, the 2015–2016 cohort received the 2016 SVI scores, and the 2017–2019 cohort was assigned the 2018 SVI scores. Children’s birth addresses were matched to Georgia state’s census-tract-level overall SVI score and the 4 subtheme scores from the assigned year (Figure 1).

### 2.3 Statistical analysis

The study summarized descriptive information using frequencies and proportions, including UTD status for the primary vaccine series, overall and subtheme SVI scores, sex, race and ethnicity, urbanicity, genotype, SVI year, and birth year. Urbanicity was classified using the Rural–Urban Commuting Area Codes (56). SCD genotype reflects disease severity and includes the most severe form, sickle cell anemia (HbSS or HbS/B0 thalassemia), as well as HbS/*β*+ thalassemia, and Hb SC disease. Pearson’s chi-square tests were used to assess whether there were significant differences in the distribution of these variables between the UTD and non-UTD groups.

The study used separate multivariable logistic regression models to examine the association between overall and subtheme SVI scores and children’s vaccine UTD status, adjusting for sex, race, genotype, urbanicity, and birth year. The outcome variable was child’s vaccine UTD status with 1 being UTD on the vaccine or the primary vaccine series, and 0 for not UTD. The inclusion of urbanicity, genotype, and birth year as covariates adjusted for confounding due to differences in healthcare access between urban and rural areas, the severity of SCD, and temporal trends in vaccine uptake.

Odds-ratios (ORs) are reported. Statistical significance level is defined with a *p*-value less than 0.05. All statistical analysis was performed using SAS Enterprise Guide 7.1.

## 3 Results

The study cohort includes 1,337 children with SCD (Table 1). Among the cohort, 774 children (57.89%) were UTD with the primary series by age 24 months and 563 (42.11%) were not (Table 1). Sex was evenly distributed in the entire cohort and between UTD status groups. The majority of the cohort were Black or African American. Over half of the cohort had sickle cell anemia (HbSS or HbS/B0 thalassemia), with 429 (55.43%) children in the UTD group and 327 (58.08%) in the non-UTD group. Most children had a birth address in urban areas (87.08% of the UTD group and 88.63% of the non-UTD). Chi-square tests found that birth year and SVI year were significantly associated with UTD status which suggest potential temporal influences on vaccination coverage (*p* < 0.001).

**Table 1:** Descriptive statistics for study variables by immunization UTD status.

Characteristic	Children with 24 months UTD		Children with 24 months non-UTD		*p*-value^a^
	*N*	%	*N*	%	
Overall	**774**		**563**		
Sex					0.548
Male	386	49.87%	291	51.69%	
Female	388	50.13%	272	48.31%	
Race					0.665
Black	713	92.12%	511	90.76%	
Non-Black	41	5.30%	33	5.86%	
Other/unknown	20	2.58%	19	3.37%	
Ethnicity					0.638
Hispanic	13	1.68%	13	2.31%	
Non-Hispanic	734	94.83%	508	90.23%	
Other/Unknown	27	3.49%	42	7.46%	
Genotype					0.634
Sickle cell anemia (HbSS or HbS/B0 thalassemia)	429	55.43%	327	58.08%	
HbS/β+ thalassemia	21	2.71%	15	2.66%	
Hb SC disease	257	33.20%	182	32.32%	
Other/unknown	67	8.57%	39	6.93%	
Urbanicity					0.441
Urban	674	87.08%	499	88.63%	
Rural	100	12.92%	64	11.37%	
Birth year					<0.001
2008	79	10.21%	48	8.53%	
2009	94	12.14%	44	7.82%	
2010	79	10.21%	33	5.86%	
2011	68	8.79%	29	5.15%	
2012	50	6.46%	40	7.10%	
2013	50	6.46%	35	6.22%	
2014	37	4.78%	35	6.22%	
2015	60	7.75%	47	8.35%	
2016	59	7.62%	65	11.55%	
2017	71	9.17%	69	12.26%	
2018	64	8.27%	50	8.88%	
2019	63	8.14%	68	12.08%	
Social Vulnerability Index by birth cohort					<0.001
2008–2012 (2010 SVI)	370	47.80%	194	34.46%	
2013–2014 (2014 SVI)	87	11.24%	70	12.43%	
2015–2016 (2016 SVI)	119	15.37%	112	19.89%	
2017–2019 (2018 SVI)	198	25.58%	189	33.57%	
Overall SVI score					0.677
Least vulnerable (bottom quartile)^b^	93	12.02%	75	13.32%	
Moderately vulnerable (Q2/Q3)^c^	397	51.29%	277	49.20%	
Most vulnerable (top quartile)^d^	284	36.69%	211	37.48%	
Socioeconomic status					0.909
Least vulnerable (bottom quartile)^b^	115	14.86%	87	15.45%	
Moderately vulnerable (Q2/Q3)^c^	420	54.26%	299	53.11%	
Most vulnerable (top quartile)^d^	239	30.88%	177	31.44%	
Household characteristics					0.579
Least vulnerable (bottom quartile)^b^	135	17.44%	106	18.83%	
Moderately vulnerable (Q2/Q3)^c^	349	45.09%	261	46.36%	
Most vulnerable (top quartile)^d^	290	37.47%	196	34.81%	
Racial and ethnic minority status					0.073
Least vulnerable (bottom quartile)^b^	51	6.59%	49	8.70%	
Moderately vulnerable (Q2/Q3)^c^	397	51.29%	256	45.47%	
Most vulnerable (top quartile)^d^	326	42.12%	258	45.83%	
Housing type and transportation					0.894
Least vulnerable (bottom quartile)^b^	118	15.25%	90	15.99%	
Moderately vulnerable (Q2/Q3)^c^	430	55.56%	306	54.35%	
Most vulnerable (top quartile)^d^	226	29.20%	167	29.66%	

UTD, Up-to-date; Q2, Quartile 2; Q3, Quartile3. Numbers in bold indicate significance at or below 0.05 level.

a Pearson’s chi-square tests used to compare the distribution of variables across the UTD and non-UTD groups.

b Bottom quartile is from 0 to 0.25.

c Q2/Q3 is from 0.25 to 0.75.

d Top quartile is from 0.75 to 1.

If the SVI were uniformly distributed, each quartile would be expected to contain 25% of the study cohort. However, we found a higher proportion of children with SCD have birth addresses in the most vulnerable level of the overall SVI scores and subthemes. In the overall SVI, 36.69% of children with UTD status and 37.48% of children with non-UTD status were in the most vulnerable category, compared to 12.02% of children with UTD status and 13.32% of children with non-UTD status in the least vulnerable category. Similar patterns were observed across the socioeconomic status, household characteristics, and housing type and transportation subthemes, where approximately 30% of children were in the most vulnerable category, while around 15% were in the least vulnerable category. The racial and ethnic minority subtheme showed an even higher concentration in the most vulnerable category, with 42.12% of children with UTD status and 45.83% of children with non-UTD status. This is expected, as the majority of children with SCD are Black, leading to higher scores in this subtheme.

No statistically significant relationship was observed between overall SVI scores and UTD vaccine completion for the entire primary series (Moderate vulnerability OR: 1.08, *p* = 0.674, 95% CI = 0.76, 1.53; High vulnerability OR: 0.95, *p* = 0.768, 95% CI = 0.66, 1.36) or individual vaccines even when adjusting for covariates (Table 2). However, children with moderate or high vulnerability in the socioeconomic subtheme had significantly higher odds of completing several individual vaccines. The odds of completing the poliovirus vaccine among children with moderate vulnerability and high vulnerability were 2.17 (*p* = 0.006, 95% CI = 1.25, 3.76) and 2.19 times (*p* = 0.023, 95% CI = 1.12, 4.29), respectively, the odds among comparable children with the least vulnerability. Children with moderate or high vulnerability also had higher odds of completing the MMR vaccine (Moderate vulnerability OR: 1.72, *p* = 0.031, 95% CI = 1.05, 2.81; High vulnerability OR: 1.84, *p* = 0.048, 95% CI = 1.01, 3.37), the *haemophilus influenzae* type-b vaccine (Moderate vulnerability OR: 1.99, *p* = 0.016, 95% CI = 1.13, 3.48; High vulnerability OR: 2.39, *p* = 0.013, 95% CI = 1.20, 4.76), and the hepatitis B vaccine (Moderate vulnerability OR: 2.60, *p* = 0.001, 95% CI = 1.46, 4.64; High vulnerability OR: 2.71, *p* = 0.006, 95% CI = 1.33, 5.53) compared to children with low vulnerability. However, having high vulnerability in the housing type and transportation subtheme had a marginal negative influence on completing the DTaP vaccine (High vulnerability OR: 0.64, *p* = 0.05, 95% CI = 0.40, 1.00).

**Table 2:** Multivariable logistic regression and ORs predicting UTD status for the primary series and individual vaccines.

Variables	Overall UTD		DTAP UTD		IPV UTD		MMR UTD		HIB UTD		HEPB UTD		VARI UTD		PNEUMO UTD	
	OR (95% CI)	*p*-value	OR (95% CI)	*p*-value	OR (95% CI)	*p*-value	OR (95% CI)	*p*-value	OR (95% CI)	*p*-value	OR (95% CI)	*p*-value	OR (95% CI)	*p*-value	OR (95% CI)	*p*-value
Overall SVI score^a^ (Ref = least vulnerable–bottom quartile)^b^																
Moderately vulnerable (Q2/Q3)^c^	1.08 (0.76, 1.53)	0.674	1.16 (0.79, 1.68)	0.448	1.31 (0.79, 2.11)	0.287	1.26 (0.80, 1.94)	0.308	1.29 (0.77, 2.10)	0.316	1.40 (0.83, 2.32)	0.192	1.09 (0.74, 1.58)	0.653	1.25 (0.85, 1.84)	0.247
Most vulnerable (top quartile)^d^	0.95 (0.66, 1.36)	0.768	1.05 (0.70, 1.55)	0.817	1.35 (0.79, 2.25)	0.264	1.32 (0.82, 2.10)	0.244	1.23 (0.72, 2.06)	0.445	1.54 (0.88, 2.64)	0.125	1.04 (0.70, 1.55)	0.837	1.18 (0.78, 1.76)	0.427
Socioeconomic status^a^ (Ref = least vulnerable–bottom quartile)^b^																
Moderately vulnerable (Q2/Q3)^c^	1.05 (0.72, 1.54)	0.787	1.25 (0.83, 1.88)	0.293	2.17 (1.25, 3.76)	**0.006**	1.72 (1.05, 2.81)	**0.031**	1.99 (1.13, 3.48)	**0.016**	2.60 (1.46, 4.64)	**0.001**	1.05 (0.69, 1.59)	0.812	1.32 (0.87, 2.01)	0.194
Most vulnerable (top quartile)^d^	0.99 (0.63, 1.56)	0.962	1.60 (0.97, 2.64)	0.068	2.19 (1.12, 4.29)	**0.023**	1.84 (1.01, 3.37)	**0.048**	2.39 (1.20, 4.76)	**0.013**	2.71 (1.33, 5.53)	**0.006**	0.89 (0.54, 1.48)	0.666	1.61 (0.96, 2.70)	0.071
Household characteristics^a^ (Ref = least vulnerable–bottom quartile)^b^																
Moderately vulnerable (Q2/Q3)^c^	0.99 (0.72, 1.37)	0.953	1.23 (0.87, 1.75)	0.24	0.93 (0.57, 1.51)	0.775	1.19 (0.77, 1.80)	0.429	1.02 (0.61, 1.66)	0.947	0.95 (0.56, 1.58)	0.853	0.94 (0.66, 1.35)	0.746	1.07 (0.74, 1.54)	0.709
Most vulnerable (top quartile)^d^	0.97 (0.67, 1.41)	0.886	1.03 (0.69, 1.54)	0.892	0.83 (0.46, 1.47)	0.528	1.09 (0.66, 1.79)	0.721	0.71 (0.39, 1.25)	0.237	0.82 (0.44, 1.50)	0.523	0.96 (0.63, 1.45)	0.853	0.84 (0.55, 1.28)	0.422
Racial and ethnic minority status^a^ (Ref = least vulnerable–bottom quartile)^b^																
Moderately vulnerable (Q2/Q3)^c^	1.42 (0.91, 2.20)	0.121	1.09 (0.65, 1.77)	0.739	0.90 (0.40, 1.82)	0.778	1.06 (0.54, 1.95)	0.856	0.69 (0.28, 1.48)	0.375	0.84 (0.34, 1.84)	0.691	1.54 (0.96, 2.45)	0.072	1.11 (0.66, 1.83)	0.692
Most vulnerable (top quartile)^d^	1.18 (0.74, 1.86)	0.486	0.86 (0.51, 1.42)	0.553	0.57 (0.25, 1.19)	0.156	0.70 (0.35, 1.08)	0.279	0.47 (0.19, 1.04)	0.083	0.46 (0.18, 1.02)	0.077	1.20 (0.73, 1.95)	0.468	0.93 (0.54, 1.56)	0.787
Housing type and transportation^a^ (Ref = least vulnerable–bottom quartile)^b^																
Moderately vulnerable (Q2/Q3)^c^	1.05 (0.74, 1.47)	0.795	0.93 (0.63, 1.35)	0.693	0.69 (0.39, 1.17)	0.184	0.67 (0.40, 1.08)	0.108	0.65 (0.36, 1.13)	0.139	0.64 (0.35, 1.13)	0.135	1.02 (0.70, 1.49)	0.916	1.05 (0.71, 1.53)	0.825
Most vulnerable (top quartile)^d^	0.98 (0.64, 1.47)	0.903	0.64 (0.40, 1.00)	**0.05**	0.63 (0.32, 1.20)	0.165	0.61 (0.34, 1.08)	0.092	0.57 (0.29, 1.10)	0.098	0.63 (0.31, 1.26)	0.196	1.07 (0.67, 1.70)	0.781	0.85 (0.53, 1.35)	0.501
Sex (Ref = Male)																
Female	1.05 (0.84, 1.32)	0.659	1.10 (0.86, 1.40)	0.473	1.36 (0.97, 1.92)	0.081	1.43 (1.06, 1.95)	**0.022**	1.14 (0.81, 1.62)	0.452	1.30 (0.91, 1.89)	0.155	1.10 (0.86, 1.41)	0.45	1.20 (0.93, 1.54)	0.17
Race (Ref = Non-Black)																
Black	1.20 (0.70, 2.05)	0.514	1.06 (0.58, 1.89)	0.84	1.29 (0.56, 2.70)	0.524	0.75 (0.32, 1.59)	0.482	1.03 (0.41, 2.29)	0.944	0.94 (0.35, 2.17)	0.889	1.04 (0.56, 1.89)	0.889	1.02 (0.55, 1.84)	0.951
Other	1.05 (0.47, 2.39)	0.898	0.58 (0.25, 1.35)	0.204	0.69 (0.24, 2.07)	0.498	0.27 (0.09, 0.71)	0.009	0.40 (0.13, 1.19)	0.1	0.47 (0.14, 1.50)	0.198	0.71 (0.29, 1.72)	0.443	0.44 (0.19, 1.04)	0.062
Genotype (Ref = Sickle cell anemia)																
HbS/β+ thalassemia	1.09 (0.51, 2.39)	0.826	0.83 (0.37, 1.91)	0.647	1.19 (0.40, 4.46)	0.778	0.97 (0.36, 2.95)	0.947	0.93 (0.33, 3.14)	0.903	0.98 (0.31, 3.78)	0.609	1.38 (0.57, 3.62)	0.489	1.07 (0.46, 2.67)	0.888
Hb SC disease	1.08 (0.85, 1.38)	0.531	0.98 (0.75, 1.29)	0.9	0.90 (0.62, 1.30)	0.557	0.94 (0.68, 1.31)	0.731	1.13 (0.78, 1.66)	0.538	0.90 (0.61, 1.34)	0.687	1.10 (0.84, 1.44)	0.48	0.93 (0.70, 1.22)	0.581
Other/unknown	1.34 (0.86, 2.12)	0.198	1.06 (0.66, 1.75)	0.803	1.03 (0.55, 2.08)	0.932	1.17 (0.64, 2.28)	0.622	1.28 (0.66, 2.69)	0.494	1.16 (0.58, 2.55)	0.462	1.45 (0.87, 2.49)	0.163	0.86 (0.54, 1.42)	0.549
Urbanicity (Ref = Urban)																
Rural	1.14 (0.78, 1.67)	0.497	1.44 (0.93, 2.26)	0.109	1.18 (0.64, 2.31)	0.611	1.43 (0.81, 2.62)	0.232	1.43 (0.75, 2.92)	0.299	1.32 (0.66, 2.88)	0.462	0.99 (0.66, 1.52)	0.975	1.37 (0.88, 2.19)	0.177
Birth year (Ref = 2008)																
2009	1.24 (0.75, 2.08)	0.404	1.39 (0.81, 2.43)	0.236	0.97 (0.44, 2.15)	0.949	1.29 (0.66, 2.54)	0.457	1.13 (0.51, 2.54)	0.761	0.77 (0.35, 1.69)	0.518	1.06 (0.56, 2.02)	0.861	1.13 (0.65, 1.95)	0.660
2010	1.41 (0.81, 2.45)	0.224	1.47 (0.82, 2.66)	0.196	1.06 (0.46, 2.53)	0.892	1.95 (0.91, 4.43)	0.094	1.17 (0.50, 2.80)	0.712	1.21 (0.49, 3.09)	0.683	1.47 (0.72, 3.11)	0.295	1.75 (0.95, 3.27)	0.075
2011	1.38 (0.78, 2.45)	0.268	1.31 (0.72, 2.41)	0.378	0.88 (0.38, 2.05)	0.757	0.94 (0.47, 1.88)	0.851	0.93 (0.40, 2.18)	0.867	1.01 (0.42, 2.51)	0.985	1.02 (0.51, 2.06)	0.966	1.29 (0.70, 2.39)	0.418
2012	0.76 (0.43, 1.31)	0.32	1.45 (0.78, 2.71)	0.243	1.46 (0.57, 4.02)	0.444	1.79 (0.82, 4.19)	0.157	2.15 (0.78, 6.90)	0.162	1.55 (0.58, 4.62)	0.398	0.76 (0.39, 1.51)	0.429	1.70 (0.90, 3.31)	0.11
2013	0.88 (0.50, 1.56)	0.664	1.30 (0.71, 2.44)	0.403	0.95 (0.40, 2.34)	0.912	1.24 (0.59, 2.67)	0.574	1.24 (0.50, 3.27)	0.655	1.32 (0.51, 3.70)	0.582	0.58 (0.30, 1.12)	0.103	1.18 (0.64, 2.23)	0.596
2014	0.66 (0.36, 1.19)	0.164	1.17 (0.62, 2.27)	0.63	0.97 (0.39, 2.57)	0.945	0.97 (0.46, 2.11)	0.939	1.11 (0.43, 3.12)	0.830	0.88 (0.35, 2.37)	0.795	0.31 (0.16, 0.60)	**<0.001**	1.73 (0.86, 3.60)	0.131
2015	0.77 (0.45, 1.31)	0.33	1.30 (0.73, 2.34)	0.38	1.36 (0.56, 3.44)	0.5	1.76 (0.84, 3.83)	0.141	1.01 (0.44, 2.36)	0.981	1.47 (0.58, 3.91)	0.424	0.46 (0.25, 0.84)	**0.013**	1.28 (0.71, 2.32)	0.419
2016	0.53 (0.31, 0.87)	**0.013**	1.05 (0.61, 1.82)	0.849	0.77 (0.35, 1.67)	0.515	1.29 (0.65, 2.58)	0.461	0.70 (0.32, 1.49)	0.360	1.06 (0.46, 2.51)	0.885	0.32 (0.18, 0.57)	**<0.001**	1.30 (0.74, 2.31)	0.363
2017	0.62 (0.38, 1.02)	0.06	1.20 (0.70, 2.05)	0.508	0.67 (0.32, 1.38)	0.282	1.05 (0.55, 1.99)	0.888	0.67 (0.32, 1.39)	0.290	0.70 (0.32, 1.49)	0.363	0.31 (0.17, 0.53)	**<0.001**	1.39 (0.80, 2.44)	0.243
2018	0.75 (0.44, 1.26)	0.272	1.13 (0.64, 1.99)	0.671	0.68 (0.31, 1.47)	0.330	1.10 (0.56, 2.20)	0.776	0.80 (0.36, 1.76)	0.573	0.73 (0.32, 1.64)	0.446	0.43 (0.23, 0.77)	**0.006**	1.08 (0.61, 1.92)	0.796
2019	0.56 (0.34, 0.92)	**0.023**	1.11 (0.65, 1.91)	0.704	0.87 (0.40, 1.89)	0.728	1.01 (0.53, 1.93)	0.980	0.79 (0.36, 1.70)	0.554	1.01 (0.44, 2.32)	0.384	0.31 (0.17, 0.54)	**<0.001**	1.24 (0.71, 2.17)	0.449
Model fit (AIC)																
Overall SVI score	1821.80		1585.25		984.67		1169.87		972.73		899.13		1561.65		1509.19	
SVI subthemes	1830.54		1584.04		985.49		1169.45		971.65		895.82		1566.71		1515.62	

UTD, Up-to-date; Q2, Quartile 2; Q3, Quartile3; DTAP, diphtheria, tetanus, and acellular pertussis vaccine (4 doses); IPV, poliovirus vaccine (3 doses); MMR, measles, mumps, and rubella vaccine (1 dose); HIB, haemophilus influenzae type-b vaccine (3 or 4 doses depending on vaccine product type); HEPB, hepatitis B vaccine (3 doses); VARI, varicella vaccine (1 dose); PNEUMO, pneumococcal conjugate vaccine (4 doses of 7-valent or 13-valent vaccine); OR, Odds-ratio; 95% CI, 95% Confidence interval; SVI, social vulnerability index; Ref, reference group. Numbers in bold indicate significance at or below 0.05 level.

a The overall SVI and subthemes are in two separate models.

b Bottom quartile is from 0 to 0.25.

c Q2/Q3 is from 0.25 to 0.75.

d Top quartile is from 0.75 to 1.

Females had higher odds of completing the MMR vaccine than males (OR: 1.43, *p* = 0.022, 95% CI = 1.06, 1.95). Additionally, children born in 2016 (OR: 0.53, *p* = 0.013, 95% CI = 0.31, 0.87) and 2019 (OR: 0.56, *p* = 0.023, 95% CI = 0.34, 0.92) had lower odds of completing the primary series compared to those born in 2008. Children born after 2013 had significantly lower odds of completing the varicella vaccine, with approximately 60% lower odds compared to those born in 2008 (2014 OR: 0.31, *p* < 0.001, 95% CI = 0.16, 0.60; 2015 OR: 0.46, *p* = 0.013, 95% CI = 0.25, 0.84; 2016 OR: 0.32, *p* < 0.001, 95% CI = 0.18, 0.57; 2017 OR: 0.31, *p* < 0.001, 95% CI = 0.17, 0.53; 2018 OR: 0.43, *p* = 0.006, 95% CI = 0.23, 0.77; 2019 OR: 0.31, *p* < 0.001, 95% CI = 0.17, 0.54).

## 4 Discussion

This study finds that a majority of children with SCD are born in census tracts characterized by high overall social vulnerability, as well as elevated vulnerability across all four subthemes. This finding aligns with previous literature documenting the lower socioeconomic status for this population (51, 52, 57). The presence of children with SCD in areas of elevated vulnerability to adverse events underscores the need for increased attention to this population.

Census-tract-level socioeconomic factors significantly increased the odds of UTD status for the poliovirus, MMR, *haemophilus influenzae* type-b, and hepatitis B vaccines. This finding is seemingly unexpected given that prior SVI studies have typically linked greater neighborhood socioeconomic vulnerability to lower immunization coverage (43–46). However, this might be due to differences in study populations and the vaccines evaluated. Previous SVI studies primarily focused on adult populations, whereas this study analyzed children, a group more likely to receive consistent vaccination monitoring through pediatric care, parental involvement, and public health follow-up.

When examining the literature on neighborhood socioeconomic indices and health outcomes among individuals with SCD, findings are mixed. Some studies report positive associations between socioeconomic disadvantage and worse health outcomes (58, 59) or lower use of preventive care (57), whereas others do not (51, 60, 61). One prior study (61) examining acute chest syndrome in the pediatric SCD population found that residence in a highly deprived neighborhood was associated with a lower risk of acute chest syndrome, and that community racial composition accounted for this association. Another study of children with SCD in Michigan reported a similar buffering effect of neighborhood ethnic concentration on receipt of transcranial Doppler screening (51). Greater racial homogeneity within a neighborhood may foster stronger social support networks, a heightened sense of belonging, and increased sharing of health-related information, which can mitigate adverse health outcomes associated with individual-level socioeconomic disadvantage (62). Although in our study the neighborhood socioeconomic subtheme independently affects immunization UTD status as we adjusted for the neighborhood minority subtheme, it is still possible that interpersonal support networks in areas with higher socioeconomic vulnerability contribute to the observed association with immunization status. Moreover, the positive association between neighborhood socioeconomic status and vaccine coverage may be partially explained by more frequent utilization of medical care among this population. Prior studies have shown that children with SCD from lower socioeconomic backgrounds use the emergency department (63–65) and other medical care more often. During these visits, providers would routinely review immunization records and refer patients to their primary care provider for follow-up care including administration of any needed vaccines. In addition to higher healthcare utilization among children with lower socioeconomic status, several health policies and programs that reduce financial barriers for low-income families may also contribute to higher vaccination rates. Vaccines in the primary series are covered by the federal Vaccines for Children Program, which removes cost barriers by providing free vaccines for children who are Medicaid eligible, uninsured, or underinsured (66, 67). Additionally, these findings may reflect the effectiveness of public health measures such as the Children’s Health Insurance Program in Georgia (PeachCare for Kids), where low-income families without Medicaid or private health insurance can enroll their kids to cover immunizations and regular check-ups (68, 69). Between 2008 to 2019, the percent of all eligible children participating in Medicaid or the Children’s Health Insurance Program in Georgia has been increasing from 81 to 89.1% (70). As of October 2023, about 225,000 eligible children in Georgia were enrolled in the program (71). Copays are waived for children under age 6 years (71); this would substantially alleviate the financial burden for low-income families and motivate health care utilization for younger-age children. Studies have found that children covered by Medicaid and the Children’s Health Insurance Program are more likely to receive preventive care than privately insured (72). Another potential explanation for higher vaccination rates in the low socioeconomic status group is that parents with lower education might be less likely to challenge providers and therefore have reduced vaccine hesitancy. However, literature on this relationship is mixed (73). One study documented that education is negatively related to COVID vaccination, while others found that lower education is related to higher vaccine hesitancy (74–76).

Higher vulnerability in the housing type and transportation marginally decreases the odds of completing the DTaP vaccine. This might suggest access barriers to preventive healthcare services among those living in aggregated housing types or without a vehicle, especially for vaccines that require multiple doses at different ages (30, 77). However, this result should be interpreted with caution considering its marginal statistical significance.

The decreased odds of completing the varicella vaccine after 2013 aligns with the observed increase in under-vaccination in recent years (78). Specifically, the varicella vaccine is more likely to cause hesitancy due to parental beliefs that disease severity is mild and vaccination is unnecessary (79–81). In addition, the low incidence of varicella outbreaks since the introduction of this vaccine also leads parents to underestimate the need for vaccination (79, 82–84). For parents of children with sickle cell disease, they might be more hesitant to vaccinate their child with varicella vaccine, which is a live vaccine, when their child is on hydroxyurea (82, 85).

Individuals living in low resourced areas or those who have trouble accessing healthcare services are more vulnerable during disease outbreaks and may lack sufficient resources to prevent disease. Without protection from immunizations, children with SCD are at heightened risk for severe health complications and hospitalizations that can lead to substantial medical expenses, missed school and work for parents, and loss of stable income. An U.S. study found that individuals with SCD spend an average of 5.1 days in the hospital per admission, at a cost of $7,637.95 per patient (86). This will add to the disease and financial burden already imposed on individuals with SCD (87, 88). Furthermore, individuals living in the most socioeconomically deprived areas face a higher risk of hospital readmission and inpatient mortality (59) according to a study in England. Therefore, promoting preventive care for this population is essential not only for health reasons but also for addressing broader socioeconomic and health disparities.

In addition to efforts to improve immunization rates among individuals who visit healthcare facilities, it is also important to proactively reach out to those who are not connected to care. The use of the SVI score provides opportunity for preventive care services and vaccination efforts to locate communities with barriers to accessing preventive services, such as those with high vulnerability in the housing and transportation theme. For populations at higher risk for morbidity and mortality due to infectious diseases, such as individuals with SCD, it is especially important to reach out to them to understand their situations and provide timely assistance.

### 4.1 Limitations

This study has several limitations that might influence the findings’ validity and generalizability. First, the study assigned each child with a specific year’s SVI. The extent to which that year’s SVI accurately reflects the child’s social vulnerability conditions from birth to 24 months may influence the study’s results. Further, the study used children’s birthing address to link with SVI census tract scores from birth to 24 months, and therefore does not account for potential mobility during this period. One investigation in Georgia suggested that 27% of children living with SCD and aged less than 9 years have one or two zip code changes during 2014 to 2016 (89). In addition, children’s immunization records were extracted from the state’s immunization registry, which may be subject to underreporting of children or their vaccines not being captured by the registry or human errors. This may be especially relevant for children born in rural areas with limited access to broadband technology, whose records may not be fully captured in the registry. Lastly, since this study is based on the U.S. context, its generalizability to global settings may be limited.

The SVI and its subthemes should be interpreted as measures of community-level vulnerability, which is conceptually distinct from the individual-level vulnerabilities assessed in other studies (90, 91). Previous research has demonstrated that individual-level socioeconomic risk does not necessarily align with community-level socioeconomic vulnerability (92, 93). Future research, particularly qualitative studies, that further examines individuals’ experiences and pediatric immunization coverage in low socioeconomic status areas would be valuable.

## 5 Conclusion

A greater proportion of children with SCD reside in areas with high social vulnerability. Current public health programs that expand access to preventive care for children from low socioeconomic backgrounds might have been effective in improving immunization coverage. Vaccine promotion efforts should prioritize communities with elevated vulnerability in the housing type and transportation theme, with a particular focus on promoting vaccines that require multiple doses as well as the varicella vaccine.

## Data Availability

The data analyzed in this study is subject to the following licenses/restrictions: they contain identifiable individual information. Requests to access these datasets should be directed to Georgia Department of Public Health.
